# The development of a lay health worker delivered collaborative community based intervention for people with schizophrenia in India

**DOI:** 10.1186/1472-6963-12-42

**Published:** 2012-02-16

**Authors:** Madhumitha Balaji, Sudipto Chatterjee, Mirja Koschorke, Thara Rangaswamy, Animish Chavan, Hamid Dabholkar, Lilly Dakshin, Pratheesh Kumar, Sujit John, Graham Thornicroft, Vikram Patel

**Affiliations:** 1 Sangath Centre, 841/1 Alto-Porvorim, Bardez, Goa 403521, India; 2 London School of Hygiene and Tropical Medicine, Keppel St, London WC1E 7HT, UK; 3 Schizophrenia Research Foundation Centre, R-7A North Main Road, Anna Nagar West, Chennai 600101, India; 4 Nirmitee, Near Sahayog Hospital, Sadar Bazar, Satara 415001, India; 5 Parivartan, Near Sahayog Hospital, Sadar Bazar, Satara 415001, India; 6 Health Service and Population Research Department, Institute of Psychiatry, Kings College London, 6 De Crespigny Park, London SE5 8AF, UK

**Keywords:** Low and middle income countries, India, Community care, Mental health, Schizophrenia

## Abstract

**Background:**

Care for schizophrenia in low and middle income countries is predominantly facility based and led by specialists, with limited use of non-pharmacological treatments. Although community based psychosocial interventions are emphasised, there is little evidence about their acceptability and feasibility. Furthermore, the shortage of skilled manpower is a major barrier to improving access to these interventions. Our study aimed to develop a lay health worker delivered community based intervention in three sites in India. This paper describes how the intervention was developed systematically, following the MRC framework for the development of complex interventions.

**Methods:**

We reviewed the lierature on the burden of schizophrenia and the treatment gap in low and middle income countries and the evidence for community based treatments, and identified intervention components. We then evaluated the acceptability and feasibility of this package of care through formative case studies with individuals with schizophrenia and their primary caregivers and piloted its delivery with 30 families.

**Results:**

Based on the reviews, our intervention comprised five components (psycho-education; adherence management; rehabilitation; referral to community agencies; and health promotion) to be delivered by trained lay health workers supervised by specialists. The intervention underwent a number of changes as a result of formative and pilot work. While all the components were acceptable and most were feasible, experiences of stigma and discrimination were inadequately addressed; some participants feared that delivery of care at home would lead to illness disclosure; some participants and providers did not understand how the intervention related to usual care; some families were unwilling to participate; and there were delivery problems, for example, in meeting the targeted number of sessions. Participants found delivery by health workers acceptable, and expected them to have knowledge about the subject matter. Some had expectations regarding their demographic and personal characteristics, for example, preferring only females or those who are understanding/friendly. New components to address stigma were then added to the intervention, the collaborative nature of service provision was strengthened, a multi-level supervision system was developed, and delivery of components was made more flexible. Criteria were evolved for the selection and training of the health workers based on participants' expectations.

**Conclusions:**

A multi-component community based intervention, targeting multiple outcomes, and delivered by trained lay health workers, supervised by mental health specialists, is an acceptable and feasible intervention for treating schizophrenia in India.

## Background

Schizophrenia is a severe mental illness characterised by chronic or relapsing symptoms [1]. Although a low prevalence disorder of 4.6 per 1000 population [2], it is amongst the top ten leading causes of disability worldwide, resulting in enormous economic and social costs for families and public health systems [3]. Although the treatment of schizophrenia should ideally comprise a combination of medication and psychosocial interventions, with a strong community orientation and involvement of family caregivers [4,5], the vast majority of affected individuals in low and middle income countries (LMIC) do not receive such comprehensive care [6]. An important reason for this 'treatment gap' is the lack of mental health specialists in LMIC [7]. "Task sharing" with appropriately trained and supervised lay or community health workers has become a widely adopted strategy to improve access to evidence-based mental health care interventions in LMIC [8].

This paper describes the development of a lay health worker led community based intervention for people with schizophrenia and their caregivers, following the steps prescribed by the Medical Research Council (MRC) framework, UK, for the development of complex interventions [9]. This process involved the understanding of the burden of the illness and limitations of current management approaches in LMIC; identification of the evidence base for the proposed intervention; the development of a theoretical model based on this evidence; and formative and piloting studies of the acceptability and feasibility of this model with a sample of people with schizophrenia and their caregivers. The intervention developed as a result of this process is currently being evaluated in a randomized controlled trial- the COPSI (Community Care for People with Schizophrenia in India) study (ISRCTN 56877013) [10].

### Intervention development

#### Phase 1: Identifying gaps in usual care

The study was conducted in three sites in India. The first was Goa, a state in western India with a population of over 1 million, nearly 50% of whom live in urban areas. The main sources of revenue in Goa include tourism and agriculture. The literacy rate is over 80%. The second site was Satara, a district in western Maharashtra with a population of nearly 3,000,000, 18% of whom live in urban areas. The literacy rate in Satara is also over 80%. Major sources of employment include agriculture, sugar industries and textiles. The third site was a catchment area of three rural blocks in the Kanchipuram district of northeastern Tamil Nadu (TN). The combined population of these blocks is about 700,000 and the average literacy rate is 70% with agriculture being the main occupation [11].

We assessed the infrastructure, service providers and treatments available at the settings of our collaborating partners at each site. In TN, observations of people with schizophrenia and their caregivers were conducted at a community mental health clinic and in Goa, at premises of private psychiatrists. Four psychiatrists in Goa were interviewed and asked to complete questionnaires recording treatments they had provided for 10 people with schizophrenia each. A summary of the findings was presented to the collaborating psychiatrists in Satara and their views about the similarities and differences in their setting were recorded, to define usual care across the study sites.

In all sites, treatment was provided in health care facilities, largely by psychiatrists with limited or no roles played by other health care providers. People with schizophrenia travelled long distances and waited considerable periods of time to see the doctor. Consultations were between 15 and 45 minutes and individuals were provided with antipsychotic and other psychotropic medicines (sedatives, antidepressants etc.). People presenting for the first time were reviewed once or twice in a month until symptom remission/improvement whereas those already in care were reviewed on an 'as needed' basis (once in a month to once in three months). In addition to medication, individual psychiatrists provided varying degrees of psycho-education: advice on adherence; advice on diet, lifestyle and health; and referral to any rehabilitation services available in the vicinity. No service was provided outside the facilities and the primary focus was on symptom reduction with pharmacological treatments. There was limited focus on long-term social or occupational outcomes and little effort made to manage stigma and discrimination experienced by the people with schizophrenia and their caregivers. Phase 1 thus showed that 'usual care' mostly addressed 'positive' symptoms of schizophrenia through medication prescribed by psychiatrists in health care facilities, reinforcing the need for community based interventions that target improving the overall quality of life of people with schizophrenia and their caregivers.

#### Phase 2: Identifying intervention components and modelling their impact

Community based interventions for people with serious mental illness, in both high income countries and in LMIC, for example, emphasising community mental health teams, case management, psycho-education, family interventions and rehabilitation have succeeded in reducing disabilities, decreasing hospitalisation rates and improving adherence, social integration and employment [12-19]. However, some of these models, for example, community mental health teams, require considerable investments of financial and human resources and may have limited feasibility in countries such as India. Thus, there was a need for developing a community based intervention whose components would be evidence-based and which could be delivered by lay community health workers.

**Figure 1:**
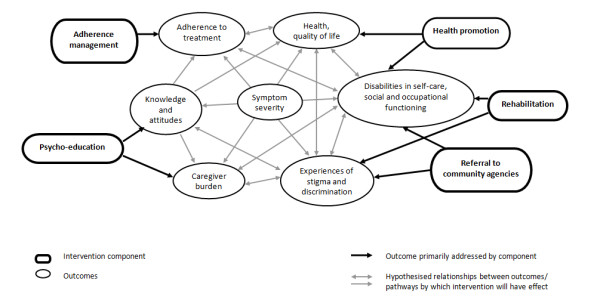
**Modelling of Intervention components and pathways to outcomes**.

We selected our initial community based intervention (CBI) components based on two sources of evidence. First, we conducted reviews of interventions for schizophrenia in LMIC [4,20]. Second, we were influenced by the experiences of a quasi-experimental study in rural India [19], in which a community based rehabilitation model was delivered by locally recruited, non-specialist health workers, in collaboration with families, the local community and psychiatrists. The defining features of this intervention were its use of a combination of evidence-based strategies; its emphasis on utilising available community resources; and its focus on improving awareness, promoting social inclusion and vocational rehabilitation. Results showed that the intervention significantly reduced symptoms and disabilities, compared to facility based care, with adherence being a strong predictor of outcomes. Based on this evidence, we identified a number of core components for our model: ***psycho-education ***(providing information about the illness); ***adherence management ***(increasing regular and correct use of medication through adherence strategies and side-effect management); ***rehabilitation ***(improving functional abilities by providing social, vocational and other skills-training, and scheduling of daily activities); and ***referral to community agencies ***(enhancing community support by improving knowledge of and access to disability benefits, employment agencies and social welfare organisations). Given the burden of co-morbid physical and mental health conditions associated with schizophrenia [21], we added an additional component on ***"health promotion"***, to this model; this focuses on improving the health of people with schizophrenia through better self-care, appropriate diet and lifestyle, and stress and anger management. In addition to these components, we also targeted the involvement of the person's family in the intervention by employing specific strategies, for example, involving them in planning the treatment; by educating them about illness and providing them with information about treatments and relapse recognition and prevention; helping them cope with difficult symptoms; and involving them in managing adherence. Intervention components such as psycho-education and family interventions have also been found to be effective in high income countries [22,23]. The modelling of components and pathways to desired outcomes is shown in Figure 1.

Based on these experiences, three principles guided our understanding of the delivery of the intervention. The first was that the intervention would be a collaborative effort and would be provided by a team comprising three key sets of persons. Firstly, the Community Lay Health Workers (CLHWs), recruited from locally available human resources (having at least 10 years of schooling) with no prior training in mental health, would be responsible for delivering the community based, non-pharmacological components of the intervention. Each CLHW would work with about 15-25 people with schizophrenia and their caregivers. Secondly, intervention coordinators (mental health specialists such as psychiatric social workers) would supervise the CLHW's and be responsible for the overall development and coordination of the intervention at their respective sites, including administrative support, training of the CLHWs and quality assurance. Finally, the treating psychiatrists would provide clinical leadership and supervision, the necessary pharmacological treatments and be involved in treatment planning and implementation. Treating psychiatrists were also involved in the process of developing the intervention, for example, the development of the research questions for the formative and piloting research stages, through their participation in monthly team meetings.

The second principle was that CBI would focus on creating an enabling environment for people with schizophrenia and their caregivers to acquire skills needed to plan for recovery. The emphasis would be on reducing both symptoms and disability, on improving knowledge and skills necessary to manage the illness, enhancing social inclusion and vocational functioning and reducing experiences of stigma and discrimination.

The third principle was that the intervention would be flexible and that the selection and delivery of the specific components would be guided by the unique needs of each individual and his/her family, identified through a structured needs assessment and responding to changes over time.

We then organised the intervention into three phases with a structured format to suit the design of the randomized controlled trial. The first "intensive engagement phase" (0-3 months) would involve weekly home based sessions with a focus on engagement, building a therapeutic alliance, needs assessment for treatment planning and the delivery of components identified as priorities through the needs assessment process. This would be followed by the "stabilisation phase" (4-7 months), where progress would be reviewed every 2-4 weeks, to ensure that the gains from the engagement phase are maintained, along with specific focus on rehabilitation, health promotion and continued psycho-education. The final, "maintenance phase" (8-12 months) would involve monthly sessions with a focus on resolving unmet needs, relapse prevention, coping strategies to deal with stigma and discrimination and social re-integration. At the end of this phase, the intervention would be terminated and the responsibility of follow-up care transferred back to the treating psychiatrists. The CLHW would be expected to conduct an average of 22 home based sessions in total, over 12 months.

#### Phase 3: Evaluating the acceptability and feasibility of intervention components

The goals of this phase were to conduct formative case studies to: (i) describe explanatory models and impact of the illness to refine the content of psycho-education and other components of the package; (ii) explore experiences of current care and unmet needs of users to refine the goals and content of CBI; and (iii) to evaluate the acceptability and feasibility of individual components and delivery mechanisms. We carried out in-depth interviews (IDIs) [24] with 32 people with schizophrenia and 38 primary caregivers recruited from psychiatric services in two study sites (Goa and TN) between September 2008 and June 2009. Assent for participation in interviews was obtained by the treating psychiatrists. Informed consent was assessed independently for people with schizophrenia and caregivers by researchers. Interviews were conducted in homes or at clinics and were audio-taped. They were transcribed and translated and then analysed using thematic analysis [25]. The study was approved by the Institutional Review Boards at Sangath and SCARF before commencement.

Briefly, findings showed that people with schizophrenia and caregivers used a variety of labels to describe the illness (ranging from diagnostic terms i.e., "schizophrenia" to less specific terms pertaining to the body i.e., "nerve problem"), and attributed the illness to stress, trauma, childhood experiences, personality factors, hereditary causes and supernatural phenomena. Both groups faced considerable burden in diverse areas of daily living related to activity, work, and social and emotional functioning as a result of the illness. People with schizophrenia experienced low self-esteem, and both those individuals and their caregivers desired to conceal the illness, and anticipated and faced discrimination from others. Current or past treatments had resulted in reduced symptoms and improvement in self-care and daily functioning, but the individuals continued to be troubled by symptoms, experienced side effects and expressed that needs regarding overall quality of life were largely unmet. A minority reported that their psychiatrists did not provide them with adequate information regarding the illness and complained of medication being expensive and of the travel and time involved in seeking treatment at facilities. A third of individuals were non-adherent with medication. In general, participants were willing to receive the CBI but recognised drug treatment as essential. They emphasised scheduling of home visits at convenient dates or timings and the need for family involvement. Some caregivers expressed concerns of having conflicting commitments (for example, jobs) that would not enable them to be present during CLHW visits. There were also concerns of home visits leading to the disclosure of illness or resulting in gossip or ridicule. Two caregivers suggested concealment of the identity of the CLHW and one was prepared to receive CBI only if it was offered away from home. Participants in Goa either had no gender preference or preferred CLHWs of the same gender as the person with schizophrenia whereas most TN participants, both male and female, preferred female CLHWs. Participants expected CLHWs to be "understanding", have good communication skills and be "friendly", "patient", "calm", "kind" and "polite" while interacting with the person with schizophrenia. In general participants wanted the CLHW to be educated (though expectations of education levels were lower in TN), but what was most important in both sites was for CLHWs to have experience with or knowledge of the subject matter.

The formative case studies confirmed that the CBI components were appropriate, relevant and important for people with schizophrenia and caregivers. However, experiences of stigma and discrimination that emerged were not sufficiently dealt with and a number of strategies were added to specifically address these factors. ***Firstly***, psycho-education was expanded by including more information to address myths about the illness; emphasising concepts of the illness being 'like any other'; providing information about recovery and what people with schizophrenia can do to enable this; and highlighting case-stories of positive outcomes. ***Secondly***, opportunities were introduced for discussing ways to cope with or respond to negative reactions from others. ***Thirdly***, CLHW received training to act as positive role models in their interactions with the family. ***Fourthly***, people with schizophrenia and caregivers were encouraged to make more informed choices about whether they wished to disclose the illness to others by discussing possible advantages and disadvantages of disclosure to specific people and conducting role plays to practice such disclosure. Furthermore, the publication of the findings of the four year follow-up study of the cohort of participants from rural India around the same time [26], led us to add the component of self-help initiatives to the intervention as these had been shown to independently predict favourable outcomes. Self-help groups comprising of affected individuals and their caregivers, or other means of peer support, were an important forum to address feelings of isolation and low self-esteem and for exchanging information (positive coping strategies for example) and expanding social networks.

We acknowledged that fear of illness disclosure if CBI was provided in homes could act as a significant barrier to intervention delivery for some people with schizophrenia. This caused us to re-examine the meaning of 'community' care, which had so far been synonymous with 'home based' care, and to consider alternative places that could be potential settings for delivering CBI for those families. We also minimised the possibility of illness disclosure by training the CLHW to specifically discuss with families strategies to prevent this, such as explanations to provide to neighbours if they were questioned about the purpose of their visit. Efforts to maximise family involvement emphasised scheduling visits at their convenience (for e.g. during weekends or in the evenings) and informing them well in advance of proposed visits.

#### Phase 4: Piloting the operational delivery of the intervention

Piloting sought to: (i) identify and address barriers to intervention delivery; (ii) further improve the acceptability and feasibility of specific components; (iii) identify barriers to the engagement of the person with schizophrenia in the intervention; and (iv) monitor the quality of intervention delivery. In keeping with the needs of the three month timeframe for the piloting exercise, we modified the intervention delivery guide to a compressed version incorporating 10 sessions over 3 months, focusing specifically on the engagement and collaborative treatment planning processes; and the delivery of the specific components of psycho-education, health promotion, adherence management and rehabilitation.

We recruited our intervention coordinators, i.e., psychologists or psychiatric social workers, by placing advertisements in the organisations' websites and in national newspapers. We recruited CLHWs by placing advertisements within the partnering organisations and in local newspapers, contacting employment agencies and NGOs for possible candidates (Goa and Satara), and approaching the local government (TN). The criteria for recruitment of CLHWs were: having at least 10 years of schooling (TN), or a graduate degree in any stream other than mental health (Goa and Satara), and commitment to helping people with schizophrenia. We interviewed recruited candidates to assess their expression of verbal ability, commitment to work, willingness to work flexible hours including holidays, and ability to travel independently for home visits. Selected candidates were trained for about 40-50 days (depending on the needs of the site). The training was conducted in local languages, by a team of persons comprising psychiatrists, psychologists, and social workers, with some sessions being conducted by other specialists (for example, dietitians). Training covered an introduction to schizophrenia, principles and methods of providing care for people with schizophrenia, the principles of the intervention, the overall structure of the COPSI program, and the specific intervention components. We used a variety of methods: lectures, role plays, group discussions, movies, short films and documentaries, quizzes, dramas, debates and games. At the end of every training module, we assessed the trainees on the following-their knowledge regarding and understanding of topics, communication skills, overall participation and team skills, through a combination of assessment methods-written tests, role plays and oral quizzes, and selected only the ones showing the most rigorous performance. Contextual variations in participants' expectations of CLHWs, identified from the formative case studies, were incorporated into their recruitment, selection and training procedures, for example, by recruiting CLHWs of both genders in Goa and Satara, and only female CLHWs in TN; in choosing people with higher levels of education (undergraduates) as CLHWs in Goa; by including additional training on positive attitudes towards schizophrenia (showing documentaries or facilitating contact with people with schizophrenia and their caregivers); and by training CLHWs specifically on desired qualities (friendliness, empathy etc.). A treatment manual was developed for the CLHWs, consisting of essential information for delivery of the CBI. It was designed for people with no previous experience of mental illnesses and had non-technical language with appropriate illustrations, examples and exercises.

Participants were recruited from all 3 project sites between March-October 2009 following the same recruitment procedures as that of the previous phase. The intervention was provided only if both the individual and the primary caregiver consented. The intervention coordinators at each site made appropriate allocations of consented participants to CLHWs (ensuring equal case load and as far as possible, in Goa and Satara, matching for gender). CBI was delivered by trained CLHW's and supervised by onsite visits by the coordinator, meetings with the psychiatrists, and group discussions with CLHWs. Evaluation included monitoring process indicators; analysis of case notes and supervision records; semi-structured interviews with 16 people with schizophrenia and 16 caregivers by researchers independent of the CBI team; interviews with the psychiatrists and a focus group discussion with the CLHWs.

##### Participation with CBI

Of the 71 people with schizophrenia who were referred for the piloting of the intervention, 4 were not contactable. 24 of the 67 families who were contacted refused participation, mostly because they were "not interested" and did not think CBI would be "useful" or provide any additional value or because symptomatic people with schizophrenia were suspicious and hostile and denied having a problem for which they required help. Additionally, in TN, participants feared that home visits were for attempts at religious conversion to Christianity (as this was not uncommon in this setting) or because they feared that accepting the intervention would place them under obligation for receiving such services in the future. In Goa, there were concerns of how CBI was related to usual care owing to the differences in the understanding of the intervention by the treating psychiatrists. One psychiatrist, for example was concerned that by encouraging the person with schizophrenia to receive CBI, he would be violating ethical considerations of patient "confidentiality".

Out of the 43 people who consented, only 30 received the intervention. This was because participants were thereafter not contactable despite repeated attempts (for example, were not available at home or had moved residence); or became symptomatic (for example, had to be admitted to inpatient care). In one case the caregiver passed away and in another, a caregiver withdrew consent (without giving a reason). In Goa, one caregiver who did decide to take part was initially hesitant about receiving the intervention because they feared home visits may result in neighbours and other family members getting to know of the illness; for this family, the intervention was offered in an alternative community setting. As expected, engaging with symptomatic people with schizophrenia was challenging and required multiple contacts, often during periods of reduced symptom severity.

##### Engagement of CLHWs

Families were initially wary of the CLHWs but were more accepting after realising that they were from the local community and following efforts made at rapport building. Engaging primary caregivers in treatment was not feasible in about 25% of the cases as some were employed and could not be present for sessions while others were unwilling to take up additional responsibility. The presence of other family members proved useful in such instances.

##### Acceptability and feasibility of components

Components were mostly acceptable but there were some feasibility barriers. People with schizophrenia and their caregivers felt that handouts had *"simple language" *and were *"easy to read" *but these could not be used with five participants who were not literate. Two participants did not read or use the handouts. Verbal explanations were however, acceptable and feasible in all cases. Components such as health promotion, for example on healthy diets, were not feasible for five participants whose economic circumstances limited food choices. In one case, the referral to community agencies component (for example to rehabilitation facilities) was not feasible as the person could not afford to travel to such places. CLHWs had difficulty with developing treatment plans for symptomatic people with schizophrenia; in addressing reported experiences of stigma; and with social skills training. They wanted more training in these areas, but felt that the supervision sessions had been useful.

##### Acceptability and feasibility of delivery process

Sessions were held once or twice a week for 60-80 minutes. The targeted number of sessions could not be met in cases where caregivers were not available for home visits or even if visits were jointly scheduled, failed to remember the appointment, or when participants were symptomatic and needed to be stabilised with medication before receiving the CBI components. CBI delivery also did not always follow the pre-planned structure-for example, rehabilitation and health promotion components, which had been planned for later sessions were sometimes addressed earlier because of felt need; needs assessment was not conducted for some participants until the third session to enable the CLHW to build rapport; and psycho-education sometimes took longer than expected for those who were not literate, for families with ongoing interpersonal disputes and for those having negative symptoms.

The intervention was modified in a number of ways following piloting. Psycho-education materials were modified; for example, they were formatted more clearly, simpler words were used and more pictures were added. Flip charts were developed to enhance the effectiveness of the information transfer; this was particularly useful for less literate participants. Strategies for dealing with economic barriers were formulated, for example people with schizophrenia unable to travel to rehabilitation facilities were offered limited vocational training at home. The session-by-session intervention delivery guide was made more flexible to reflect the unique needs and the logistics for each individual, while specifying the minimum acceptable number of sessions needed to deliver all the components. Refresher training was held for the CLHWs, focusing on social skills and stigma strategies. The scheduling of appointments at the convenience of caregivers was re-emphasised and reminders were given to families prior to home visits, to avoid cancellations. For people who were symptomatic, referral for pharmacological treatment was the priority- and CBI was offered after symptomatic improvement.

It appeared that some participants and treating psychiatrists saw CBI and usual psychiatric out-patient care as separate treatments. Consequently, we strengthened our links with the psychiatrists by emphasising their role as overall clinical team leaders, by providing them with a manual explaining their responsibilities, and requiring the CLHW to report regularly to the psychiatrist about their patients. We modified the name of the intervention to "*Collaborative *Community Based Care" (CCBC), as this was felt to better communicated the collaborative nature of our intervention delivery, and we introduced flip charts during the time of informed consent to pictorially depict this collaboration. Supervision protocols were developed for ensuring quality standards in intervention delivery. These consisted of the following methods: (i) ***onsite supervision***- the intervention coordinator to accompany the CLHWs on at least 10% of their home visits to observe the process of engagement and the delivery of CBI components; (ii) a ***quarterly review ***to assess the progress made in meeting needs for the individual and to plan for the activities for the next quarter, involving all members of the treating team; (iii) ***fortnightly review with the psychiatrist ***wherein the CLHWs and the intervention coordinator review individual clinical state and treatment plans; at these reviews, the coordinator would also discuss specific issues that may have risen, such as difficulties in engaging family members or risk for suicide, and seek advice regarding these; and (iv) ***monthly group meetings ***i.e. all CLHWs meet with the intervention coordinator for discussions on their work, with emphasis on identifying common difficulties faced in delivery and group problem solving, and on addressing personal issues and concerns experienced by the CLHW.

## Discussion

The COPSI study aimed to develop and evaluate a community based intervention delivered by community based lay health workers working in collaboration with mental health specialists. This paper describes how the intervention was developed, following the MRC framework for complex interventions, to ensure that the intervention was evidence-based and adapted to suit contextual factors.

We began by identifying unmet needs and gaps in usual care; this showed that there was a pressing need for community based interventions to address diverse needs of the person with schizophrenia and their caregivers and for locally acceptable and affordable models of delivering care in LMIC. Following this, a community based intervention was modelled on the basis of evidence-based components aimed at addressing unmet needs. The subsequent formative and piloting stages sought to assess the acceptability and feasibility of these components and their delivery by CLHWs. The intervention was then modified in a number of ways, notably by: (i) adding treatment components, self-help initiatives and strategies to address stigma; (ii) clarifying and strengthening the collaborative roles of service providers; (iii) addressing the barrier of illness concealment; (iv) incorporating a multi-level supervision protocol for quality assurance and; (v) identifying appropriate criteria and procedures for recruitment, selection and training of CLHWs.

**Figure 2:**
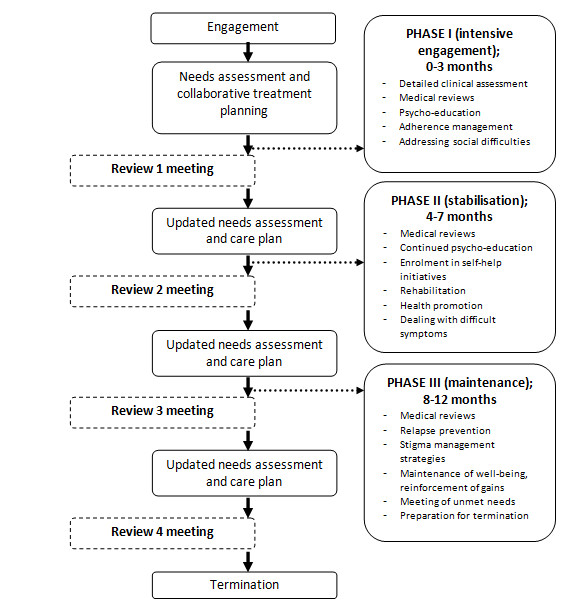
**The Collaborative Community Based Care (CCBC) delivery process**.

**Figure 3:**
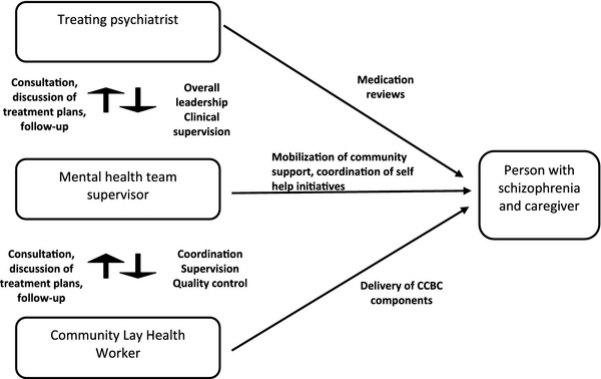
**The Collaborative Community Based Care (CCBC) mental health team**.

The final intervention comprises, in addition to usual Facility Based Care (FBC), the following components (engagement and collaborative treatment planning, psycho-education, adherence management, health promotion, rehabilitation, referral to community agencies, self-help initiatives, strategies to reduce stigma and discrimination, supervision and quality assurance, and termination) to be delivered in three phases spread over 12 months (Table 1, Figure 2). Intervention delivery comprises a 3-tier team: community lay health workers, mental health team supervisors (i.e. intervention coordinators) and psychiatrists, with clearly defined roles and responsibilities for each (Figure 3). For the purpose of the proposed randomised controlled trial, participants from TN will be identified from the community (being likely to be partially or completely untreated) and referred to a community mental health clinic for diagnosis and treatment. In Goa and Satara, participants will be those presenting for treatment at outpatient psychiatric care. Persons will be eligible to participate if they meet the ICD-10 diagnosis of schizophrenia, present with moderate or severe symptoms, and have had the Illness for at least 12 months. In each site, patients will be randomly allocated to either CCBC with FBC or FBC alone and outcomes will be compared after 12 months. Details of the trial protocol are presented elsewhere [10].

**Table 1:** The final Collaborative Community Based Care (CCBC) model

Intervention component	Specific actions	When delivered
Engagement and collaborative treatment planning	■ Building a trusting professional relationship with the individual and the key caregivers based on genuineness, respect and empathy ■ Engaging the caregiver in the intervention by encouraging their participation and providing support ■ Exploring and recording of needs and priorities of individuals and their caregivers through a structured needs assessment ■ Detailing and responding to social difficulties faced by the caregivers ■ Developing a treatment plan in collaboration with individuals, caregivers and treating psychiatrists	Specific focus in the intensive engagement phase, with the needs assessment repeated at the end of every 3 months

Medical reviews	■ Providing pharmacological treatment ■ Providing information on medications and stressing need for adherence ■ Referring acute episodes or relapses to inpatient care	Specific focus in the intensive engagement phase, and continued throughout 12 months

Adherence management	■ Understanding adherence related beliefs and stressing the need for adherence ■ Providing information about medications (benefits and side effects) ■ Maximising family support in monitoring ■ Making treatments accessible for non-adherent people by accompanying them on clinical visits or bringing home regular supplies of medications ■ Implementing adherence strategies such as use of incentives, aids (e.g. reminders, pill boxes) or changing doses/medicines ■ Side-effect management	Specific focus in the intensive engagement phase, and continued throughout 12 months

Psycho-education (for stigma actions please see below)	■ Providing information about schizophrenia, (medications, dealing with difficult symptoms, relapse prevention) for both people with schizophrenia and their caregivers	Specific focus in the intensive engagement phase, and continued throughout 12 months

Health promotion	■ Providing information and advice on healthy diets ■ Encouraging healthier lifestyle (e.g. physical exercise, stopping smoking) ■ Referring people with physical health problems to physicians ■ Helping reducing stress and anger by recognising triggers and teaching coping strategies (e.g. relaxation exercises, peaceful imagery)	Specific focus in the stabilisation phase, and continued as necessary

Rehabilitation	■ Improving self-care ■ Improving functioning in Activities of Daily Living (ADL) ■ Enhancing coping with distressing symptoms by using positive coping strategies (e.g. recreation, keeping busy) ■ Encouraging work at home or elsewhere by teaching prevocational (e.g. organisational ability) and vocational skills (e.g. computer skills) ■ Improving social interactions through social skills training ■ Encouraging attendance to community activities and resuming roles in society	Specific focus in the stabilisation phase, and continued as necessary

Referral to community agencies	■ Providing information on government schemes for disability benefits ■ Enlisting support of the local government and employers for providing employment opportunities ■ Improving access to employment opportunities through referrals to vocational and rehabilitation centres	Specific focus in the intensive engagement (while responding to social difficulties) and stabilisation phases, and continued as necessary

Self-help initiatives (meetings of affected persons/caregivers)	■ Sharing of common experiences ■ Exchanging of useful information, e.g. positive coping strategies ■ Emphasising emotional support ■ Facilitating forming of social relationships	Specific focus in the stabilisation phase, and continued as necessary

Strategies to deal with stigma and discrimination	■ Providing accurate information about the illness to dispel myths ■ Emphasising concepts of 'it's nobody's fault' or 'illness like any other' ■ Emphasising the possibility of positive outcomes ■ Addressing low self-esteem by identifying strengths and building them ■ Exploring likely outcomes of illness disclosure along with potential advantages and disadvantages of disclosing ■ Discussing ways of responding to and coping with discrimination from others	Specific focus in the maintenance phase

Supervision and quality assurance	■ For individual cases, onsite supervision by the mental health team coordinator; quarterly reviews by the whole team; and fortnightly reviews with psychiatrists. ■ For overall quality assurance and support to CLHWs, monthly meetings with the whole team	Initiated in the intensive engagement phase and continued till termination

Termination and transfer of care	■ Reviewing clinical state and treatment progress ■ Introducing strategies for long term maintenance of overall health and emotional wellbeing and for preventing relapses ■ Emphasising links with community agencies and follow up of activities to minimise experiences of stigma and discrimination ■ Formal transfer of care back to treating psychiatrists	At the end of 12 months

The COPSI trial is an important addition to scarce literature on the methodology for the development of complex interventions for severe mental disorders in low resource contexts. By following a systematic methodology, our original intervention was considerably modified in many ways and we believe that we have anticipated and addressed barriers which may have compromised the effectiveness of the intervention in its formal evaluation in a trial.

Overall, our study demonstrates that the delivery of a community based intervention by locally available, non-specialist human resources in collaboration with specialists is an acceptable and feasible approach for reducing the treatment gap for schizophrenia in LMIC.

## Abbreviations

LMIC: Low and middle income countries; COPSI: Community care for people with schizophrenia in India; TN: Tamil Nadu; CBI: Community based intervention; CLHW: Community lay health workers; CCBC: Collaborative community based care; FBC, Facility Based Care.

## Competing interests

The authors declare that they have no competing interests.

## Authors' contributions

MB was the coordinator of the study at the Goa site, developed protocols for the study, supervised data collection, carried out the analysis, and drafted the manuscript. SC was the overall trial coordinator of the study and helped with drafting the paper. MK coordinated the collection of data on stigma, analysed the data, developed strategies for addressing stigma in the final intervention and wrote the sections on stigma findings. HD and AC were the coordinators of the study at the Satara site. PK and LD were the mental health coordinators for Goa and Satara; and TN, respectively. SJ was the coordinator of the study at the TN site. TR, GT and VP were the Principal Investigators; they designed the study and helped draft the manuscript. All authors read and approved the final manuscript.

## Pre-publication history

The pre-publication history for this paper can be accessed here:

http://www.biomedcentral.com/1472-6963/12/42/prepub
